# Platelet‐rich plasma‐derived extracellular vesicles: A superior alternative in regenerative medicine?

**DOI:** 10.1111/cpr.13123

**Published:** 2021-10-05

**Authors:** Jiuping Wu, Yingxin Piao, Qinyi Liu, Xiaoyu Yang

**Affiliations:** ^1^ Department of Orthopaedics The Second Hospital Jilin University Changchun China; ^2^ Hospital of Stomatology Jilin University Changchun China

**Keywords:** extracellular vesicles, platelet‐rich plasma, platelet‐rich plasma‐derived extracellular vesicles, regenerative medicine

## Abstract

Platelet‐rich plasma (PRP), due to its promising therapeutic properties, has been used in regenerative medicine for more than 30 years and numerous encouraging outcomes have been obtained. Currently, by benefiting from new insights into PRP mechanisms and the excellent performance of extracellular vesicles (EVs) in the field of tissue repair and regeneration, studies have found that a large number of EVs released from activated platelets also participate in the regulation of tissue repair. A growing number of preclinical studies are exploring the functions of PRP‐derived EVs (PRP‐EVs), especially in tissue regeneration. Here, we summarize the latest progress in PRP‐EVs as a superior alternative cell‐free therapeutic strategy in regenerative medicine, clarify their underlying molecular mechanisms, and discuss the advantages and limitations of the upcoming clinical applications. This review highlights the potential of PRP‐EVs to replace the application of PRP or even become a superior alternative in regenerative medicine.

## Graphical abstract

Platelet‐rich plasma (PRP) has been used in regenerative medicine for more than 30 years and has obtained numerous encouraging outcomes. Previous views suggested that the powerful repair ability of PRP was derived mainly from the abundant secreted growth factors. However, over the past five years, scientists found that, in addition to growth factors, a large number of EVs were also released from activated PRP to participate in the regulation of tissue repair. This review summarizes the latest reported progress of PRP‐EVs as a superior alternative cell‐free therapeutic strategy in regenerative medicine, clarifies their underlying molecular mechanisms and discusses the advantages and limitations of the upcoming clinical applications. Compared to the well‐studied PRP, PRP‐EVs exhibit more significant advantages in regenerative medicine. With the continuous advances in the understanding of the molecular mechanisms of PRP‐EVs and with more convincing clinical evidence, we believe that PRP‐EVs may replace the application of PRP or even become a superior alternative for regenerative medicine in the near future.

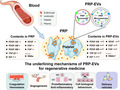

## 1 INTRODUCTION

As the human population continues to age and the incidence of degenerative and traumatic diseases continues to increase, developing therapeutic strategies to repair and regenerate damaged tissues and restore their normal functions is the most important goal of regenerative medicine.^1^ To date, the strategies of regenerative medicine have focused on using materials science and engineering techniques to develop novel biomaterials that can regulate cellular functions and activate their innate regenerative potential.^2^, ^3^, ^4^ However, in recent decades, the potential of platelet‐rich plasma (PRP) therapies has attracted considerable interest in regenerative medicine. Many high‐quality systematic reviews and meta‐analyses have summarized the encouraging results of PRP therapies in a wide range of clinical fields, including orthopaedic surgery,^5^ plastic surgery,^6^ dermatology,^7^ trichology,^8^, ^9^ cardiac surgery,^10^ maxillofacial surgery,^11^ pain management,^12^ spinal disorders,^13^ sports medicine^14^ and others.^15^, ^16^ Emerging clinical evidence suggests that PRP therapies are promising, but there remain some disadvantages and limitations that need to be considered.

In the past couple of decades, the discovery of extracellular vesicles (EVs) has been one of the most revolutionary contributions to cell biology.^17^ The ability of EVs to transport proteins, nucleic acids and lipids to target specific tissues and maintain the stability of therapeutic cargo makes EVs interesting as part of new strategies for the treatment of various diseases.^18^ Similarly, EV‐based subcellular therapies are expected to pave the way for the clinical application of regenerative medicine by overcoming the challenges of cell‐based therapies. However, most recent preclinical studies in regenerative medicine focus on mesenchymal stem cells (MSCs) as a source of EVs^19^, ^20^; relatively little attention is paid to exploring EVs derived from other cells or tissues.

Platelet‐derived EVs, as the largest part of blood EVs, have a long history of discovery.^21^, ^22^ Traditionally, due to a lack of in‐depth understanding of the functions of EVs, platelet‐derived EVs have been described as procoagulant materials released from activated platelets.^23^ Over time, this definition has been gradually accepted as they are multipurpose. Due to the great potential of PRP to promote tissue repair and regeneration, PRP‐derived EVs (PRP‐EVs) have also attracted great interest in regenerative medicine. However, as a novel promising therapy in regenerative medicine, the possible mechanisms behind PRP‐EVs and the advantages and limitations of their application still need to be further understood. (Since PRP extraction is an indispensable step in the process of extracting platelet‐derived EVs and in order to compare the role of PRP in regenerative medicine, this review uses the abbreviation “PRP‐EVs” to refer to both platelet‐derived EVs and PRP‐derived EVs.)

In this review, we systematically summarize the latest reported progress of PRP‐EVs as a superior alternative cell‐free therapeutic strategy in regenerative medicine, clarifying their underlying molecular mechanisms and discussing the advantages and limitations of the upcoming clinical applications.

## 2 DEFINITION, RATIONALES AND LIMITATIONS OF PRP IN REGENERATIVE MEDICINE

PRP is a kind of blood‐derived product that is produced via centrifugation or the apheresis process for the platelet enrichment of plasma from autologous or allogenic blood. It is characterized by a higher proportion of platelets than that of normal blood.

Since the first reviews regarding PRP applications published in 2006,^24^ many studies have provided persuasive evidence for the powerful potential of PRP to promote tissue repair and regeneration.^5^, ^6^, ^7^, ^8^, ^9^, ^10^, ^11^, ^12^, ^13^, ^14^, ^15^, ^16^ Although PRP therapies have gained increasing popularity with widespread applications in diverse regenerative fields, the understanding of the systematic scientific rationale for PRP as a biological product is far from complete. Recent studies suggest that many biomolecules from PRP are helpful in supporting PRP‐mediated tissue repair; these biomolecules include (1) primary growth factors (GFs), including platelet‐derived growth factor AA/AB/BB (PDGF AA/AB/BB), transforming growth factor beta (TGF‐β), insulin‐like growth factor 1/2 (IGF‐1/2), vascular endothelial growth factor (VEGF), epidermal growth factor (EGF), fibroblast growth factor (FGF), connecting tissue growth factor (CTGF), hepatocyte growth factor (HGF), etc.^25^, ^26^, ^27^, ^28^, ^29^; (2) cytokines, including interleukin‐1β (IL‐1β), tumour necrosis factor‐α (TNF‐α) and matrix metalloproteinases (MMPs), etc.^27^, ^30^, ^31^; (3) additional components, including fibrin, leukocytes, lysosomes, adhesion proteins (e.g., fibrinogen, fibronectin, vitronectin and thrombospondin‐1) and a series of chemokines (e.g., CCL‐2, ‐3 and ‐5, CXCL‐1, ‐4, ‐5, ‐8 and ‐12, and NAP‐2/CXCL7), etc.^32^, ^33^ Moreover, many mRNAs, microRNAs, lipids and EVs may also contribute to PRP‐mediated tissue regeneration.^34^ Based on the last few decades of research, most studies are in agreement that the underlying biomolecular rationale for PRP therapies is as a source of multiple biomolecules favouring tissue repair and regeneration.^35^

Although PRP‐based therapies are promising and have long been investigated, there remain some inconsistent views regarding their use. Some disadvantages and limitations still need to be considered. First, once released from platelets, many PRP‐derived biomolecules fail to be protected from the phospholipid membrane, which may be damaged by lytic enzymes from the extracellular environment and lose their biological activity quickly. Additionally, recent systematic reviews have revealed that the absence of unified standards for PRP preparations, classifications and clinical applications makes it more difficult to evaluate the biological functions and clinical effectiveness of PRP between different studies.^15^, ^16^ Failures in product standardization limit the scope of PRP clinical applications and the development of commercial PRP‐related products. Moreover, as one of the most comprehensively investigated blood‐derived products, the components of PRP are highly influenced by individual characteristics, including intrinsic, versatile and adaptive characteristics. The individual states of the donor, including gender, age and health status, may be present in the PRP products. The individualized features of PRP make it difficult to compare PRP therapy outcomes and may result in many studies being unable to be validated repeatedly.^15^, ^36^ Similarly, the components of PRP are as complex as those of blood and are likely more complex than many traditional pharmaceutical drugs.^15^ It is extremely difficult to explain the individual functions of different components. Thus, although no serious complications have been reported, some possible teratogenic and carcinogenic risks still need to be a focus of attention during PRP therapy. More importantly, it is well‐known that platelets lack an integral cellular structure, but cases of slight immunological rejection between allogenic individuals nonetheless exist, which may limit the clinical application of PRP to some extent.

However, current strategies face difficulties in breaking these bottlenecks. Therefore, PRP‐derived products, such as PRP‐EVs or PRP hydrogel, are expected to replace PRP in the future as more efficient and safer clinical candidates in the field of tissue repair and regeneration.

## 3 EXTRACELLULAR VESICLES: A NEW PARADIGM FOR SUBCELLULAR THERAPY

Due to the excellent proliferation and differentiation potentials, the role of progenitor/stem cells is becoming increasingly prominent and of central importance in regenerative medicine.^37^, ^38^ In recent decades, progenitor/stem cell‐based regenerative medicine strategies have developed rapidly and with many encouraging results. While promising, there have been several challenges in transitioning this strategy from bench to bedside, including immune compatibility, tumourigenicity and transmission of infections.^39^, ^40^ More importantly, diverging from early studies suggesting that the therapeutic effects of progenitor/stem cells are derived from their engraftment and differentiation at damaged tissue sites, recent studies have demonstrated that the tissue repair and regeneration by progenitor/stem cells may be driven by the paracrine activity of their secreted factors, including EVs and soluble factors.^41^, ^42^, ^43^ In the past decade, EV‐based subcellular therapies have emerged as more promising strategies to overcome these challenges associated with progenitor/stem cell‐based therapies for tissue and organ regeneration.^20^

EVs are natural nano‐sized membrane vesicles encapsulated by phospholipid bilayers, which can be secreted by various types of cells in normal or stress conditions.^44^, ^45^ According to the differences in their triggering mechanisms and biophysiological properties, EVs can be subdivided into three major types: exosomes (30–150 nm in diameter), microvesicles (50 nm–1 μm in diameter) and apoptotic bodies (100 nm–5 μm in diameter).^17^, ^46^, ^47^ In 1976, EVs were first found in the secretomes derived from platelets and defined as “platelet dust”, which are involved in bone mineralization.^48^ In the early 1980s, EVs were regarded to act as “garbage bags” with the major function of removing cellular waste.^49^ However, in the past decade, EVs have been thought of as important mediators for intercellular communication that are linked to both physiological and pathological functions. Owing to their natural properties as excellent

vectors for biological messengers and trophic factors, as well as their ability to surmount biological barriers, EVs are increasingly being studied as promising therapeutic agents.^45^ Studies are exploring EVs for potential cell‐free biotherapies for regenerative medicine and as delivery vehicles for therapeutic agents to treat cancer and inflammatory and immune diseases.^45^, ^50^, ^51^ Recently, many preclinical trials have focused on MSCs including bone marrow MSCs, adipose MSCs and umbilical cord MSCs‐derived EVs (MSCs‐EVs) to treat various forms of tissue injury, including lung injury,^52^, ^53^ kidney injury,^54^, ^55^ liver injury,^56^ central nervous system injury,^57^, ^58^ bone injury,^59^, ^60^ cartilage injury^61^, ^62^ and heart injury.^63^ However, relatively little attention has been paid to the applications of EVs derived from other cells or tissues. Over the past 5 years, benefiting from the new insights into PRP mechanisms and the excellent performance of EVs in the field of tissue repair and regeneration, PRP‐EVs have attracted growing interest as promising candidates for tissue regeneration.

## 4 HISTORICAL BACKGROUND, BIOGENESIS, ISOLATION AND FEATURES OF PRP‐EVS

As mentioned above, due to its convenient, safe and efficient properties, PRP is widely used in various clinical fields to promote tissue repair and regeneration. However, the mechanisms of PRP in regenerative medicine are not yet completely understood. Previous prevailing views suggested that the powerful repair ability of PRP is derived mainly from the abundant amounts of secreted growth factors. However, recent studies have revealed that in addition to growth factors, a large number of EVs are released after PRP activation to participate in the regulation of tissue repair.^64^, ^65^, ^66^

**FIGURE 1:**
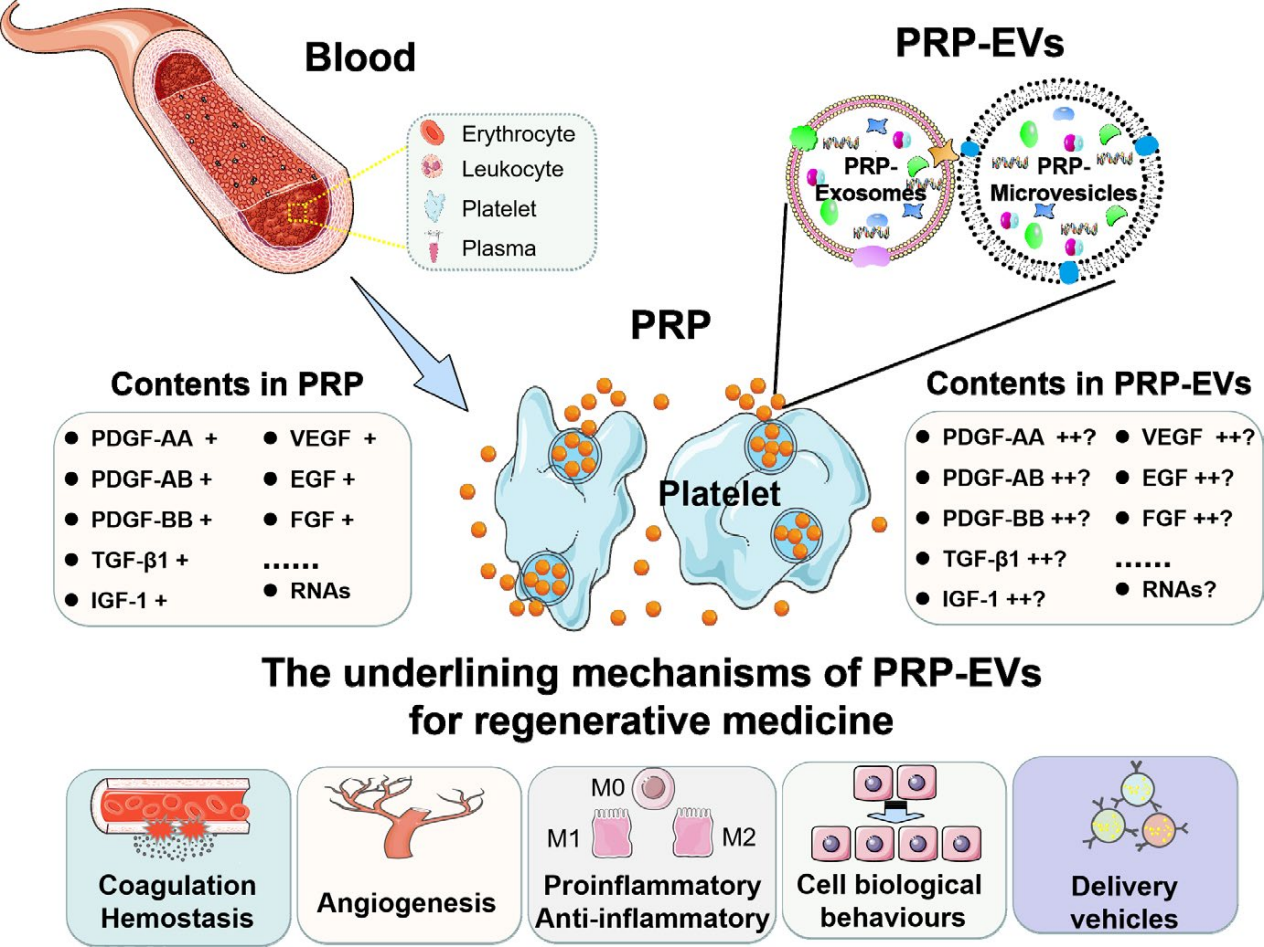
The origins and comparison of contents in PRP and PRP‐EVs. Some studies have confirmed the higher concentration of growth factors in PRP‐EVs as compared to PRP. Besides, PRP and PRP‐EVs were demonstrated with great potential in regenerative medicine

PRP‐EVs are described as a kind of subcellular vesicle released from platelets under conditions of activation, shear stress, apoptosis and injury (Figure 1).^21^ In 1967, using electron microscopic techniques, Wolf first observed these shed membrane fragments from activated platelets and described them as “platelet dust”.^48^ In 1972, Warren et al. illustrated this release process in more detail.^67^ Early studies believed that these membrane fragments shared many functional features with platelets. For instance, Sinauridze et al. demonstrated that the surfaces of these membrane fragments have a 50‐ to 100‐fold higher specific procoagulant activity than that of activated platelets.^68^ However, due to the lack of knowledge of EVs, in‐depth studies regarding their features and functions are rare. Recently, with the increased understanding of EVs, PRP‐EVs have attracted increasing attention, not only because of their excellent procoagulant activity but also because of their great potential to promote tissue repair and regeneration.^69^

**FIGURE 2:**
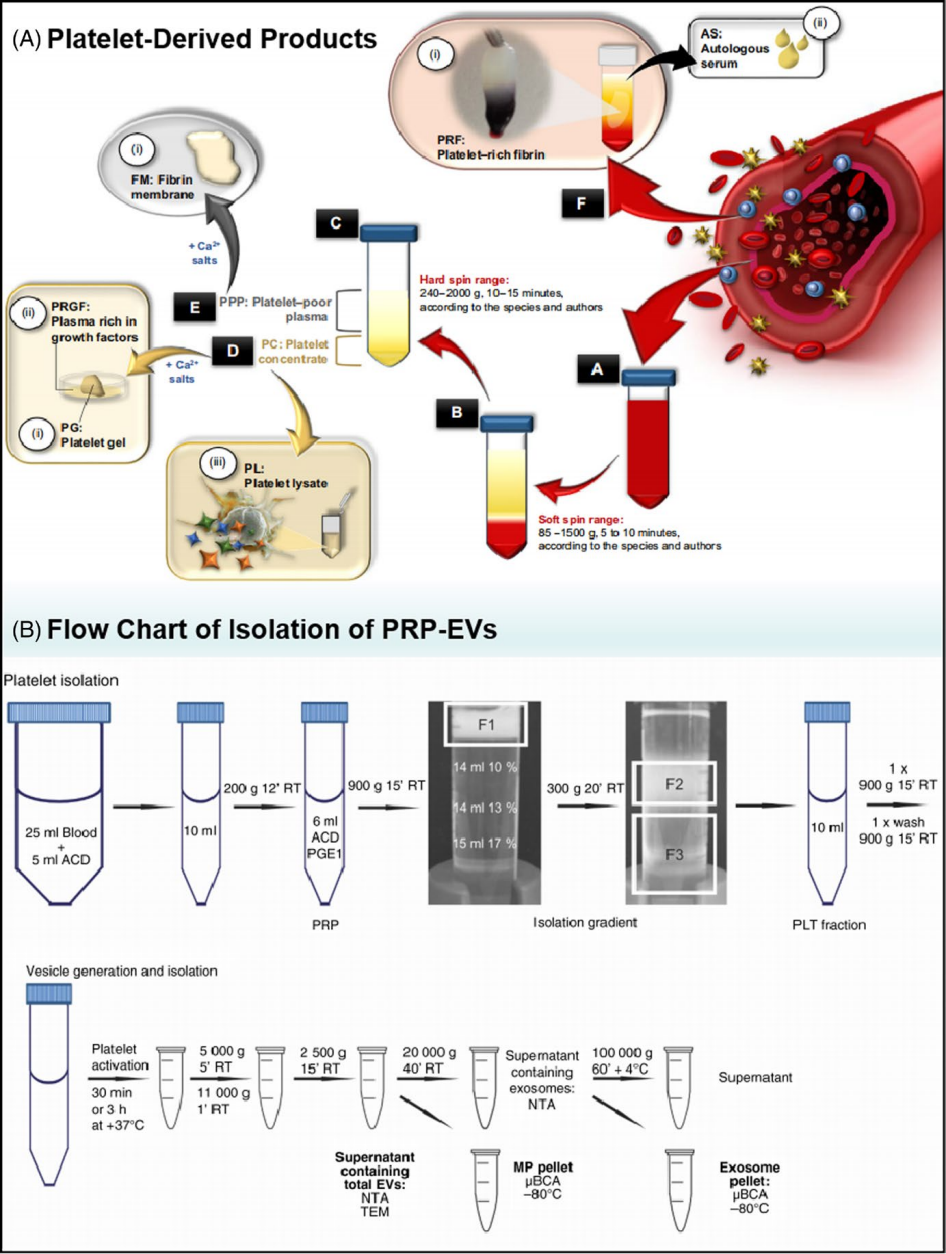
(A) The categories of platelet‐derived products and the methods of their isolation. Rich platelet‐derived products were concluded, including platelet‐rich fibrin, platelet‐poor plasma, platelet concentration, platelet gel, platelet lysate, and so on. (B) Flow chart of the isolation of PRP and PRP‐EVs. The isolation procedures of PRP‐EVs including three key steps: the isolation of PRP, activation of PRP, and isolation of PRP‐EVs^72^, ^126^

It is well known that EVs are present in a wide range of body fluids including blood, urine, saliva, CSF, amniotic fluid, breast milk and ascites.^51^ In healthy individuals, blood is one of the richest and most easily accessible sources of EVs, whereas platelet‐derived EVs contribute to the majority of blood EVs (up to 70%–90%).^22^ Recently, more accurate detection methods have also demonstrated that nearly 50% of blood EVs are derived from platelets or megakaryocytes.^70^, ^71^ Additionally, Aatonen et al. summarized the isolation procedure for PRP‐EVs, which includes three key steps: step one, PRP is extracted from whole blood; step two, PRP is activated to promote the release of EVs; and step three, PRP‐EVs are isolated by the differential centrifugation method (Figure 2).^72^

There are two subtypes of PRP‐EVs released from activated platelets: exosomes (40–100 nm in diameter), which are generated by exocytosis from the multivesicular body (MVB) and alpha‐granules, and microvesicles or microparticles (100–1000 nm in diameter), which are generated by the surface budding of the cytoplasmic membrane.^23^, ^72^, ^73^ There are many significant differences between these two types of PRP‐EVs in terms of formation mechanisms and their features (Table 1). During the generation of platelet‐derived exosomes, the plasma membrane bulges inward via endocytosis and forms the early sorting endosomes (ESEs). Then, the ESEs are matured and transformed into the MVBs under Rab5 control. In this process, intraluminal vesicles (ILVs) are formed and important proteins, RNAs and other small molecules are selectively packaged into the ILVs. Finally, the MVBs are fused with the plasma membrane to release the formed ILVs as exosomes via the endosomal sorting complex required for the transport pathway.^23^, ^48^, ^74^ However, the platelet‐derived microvesicles are generated directly by cell membrane budding. When the intracellular calcium level increases, floppase and scramblase are activated and flippase is inhibited. This leads to the reorganization of phospholipids, the breakage of bonds between the cytoskeleton and the partial degradation of actin filaments, which promote the formation and release of microvesicles.^23^, ^48^, ^75^ Moreover, some other molecular mechanisms are involved in the formation of microvesicles, including membrane curvature proteins, rho‐associated protein kinase 1, adenosine diphosphate‐ribosylation factor 6 and the contraction of actin‐myosin.^76^, ^77^

**TABLE 1:** Comparison of futures between two different subtypes of PRP‐EVs.^23^, ^72^, ^73^, ^78^, ^79^, ^85^

	PRP‐Exosomes	PRP‐Microvesicles
Size (diameter)	40–100 nm	100–1000 nm
Source	Multivesicular bodies (MVBs)	Plasma membrane
Formation mechanism	Endosomal sorting complex required for transport (ESCRT)	Shedding of membranes
Positive markers	CD63, CD9, TSG101, ALIX, and P‐selectin (limited)	GPIb (CD42b), P‐selectin, Pecam‐1, Peta‐3, and β1‐integrin
Pro‐coagulant sites	Negative	Annexin‐V, prothrombin, and factor X

**FIGURE 3:**
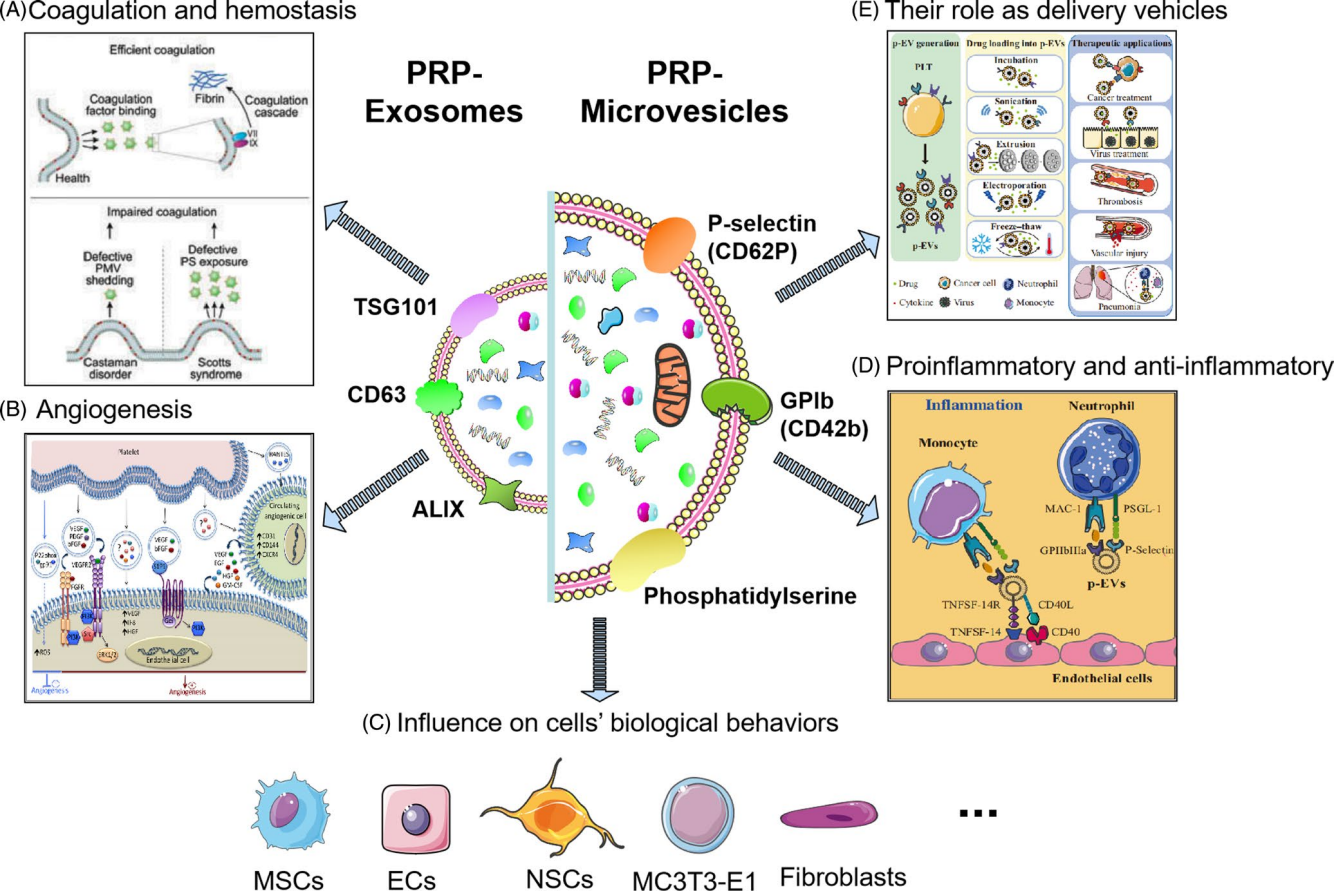
The features between two different subtypes of PRP‐EVs and five underlining mechanisms concerning regenerative medicine: procoagulant activity and hemostasis, angiogenesis, pro‐inflammatory and anti‐inflammatory properties, the influence on cells’ biological behaviors, and their role as delivery vehicles^23^, ^83^, ^127^

In general, the cargo and biological properties of EVs are defined by the types and characteristics in parental cells. According to the different methods of activating platelets, platelet‐derived microvesicles are characterized by a high expression of CD41, CD42 and phosphatidylserine (PS).^78^ Similarly, platelet‐derived exosomes are characterized by a high expression of marker proteins of exosomes, such as CD9, CD63, TSG101 and ALIX (Figure 3).^79^ However, it is difficult for the current isolation protocols to distinguish these two types of PRP‐EVs. Unless the experimental conditions can identify that the capturing vesicles are from cell membrane budding or intracellular vesicles, the current consensus is to use the umbrella term “extracellular vesicles”.^47^

Many important features are used for the unified identification of PRP‐EVs, including platelet endothelium adhesion molecule (CD31), CD41, CD42a, CD61, CD62p, CD63 and PS.^80^, ^81^, ^82^ In addition, many critical biomolecules are rich in PRP‐EVs, including growth factors, cytokines, chemokines, lipids and nucleic acids, as well as procoagulant and anticoagulant, pro‐inflammatory and anti‐inflammatory, and proangiogenic and antiangiogenic factors.^23^, ^83^, ^84^ These components and features of PRP‐EVs support their prospective therapeutic application in regenerative medicine.

## 5 PRP‐EVS IN REGENERATIVE MEDICINE

When PRP‐EVs were first discovered, their main functions were considered to be the transport of procoagulant materials, performing most of the same functions as platelets.^85^ With the continued increase in interest in PRP‐EVs, an increasing number of other functions have been confirmed. They were also demonstrated to be involved in haemostasis, vascular integrity, immunoregulation and inflammatory regulation.^86^, ^87^, ^88^ Additionally, platelet‐derived EVs have been reported to be associated with the pathological processes of some diseases, such as rheumatoid arthritis,^89^, ^90^ cancer^91^ and cardiovascular diseases.^92^ More importantly, a recent study found that PRP‐EVs have great potential in the field of tissue repair and regeneration (Table 2).^83^ For example, Guo et al. found that PRP‐EVs could improve the proliferation, migration and angiogenesis of vascular endothelial cells via the activation of the yes‐associated protein (YAP), resulting in the promotion of wound healing in a diabetic rat model.^66^ Another study by Tao et al. also demonstrated that PRP‐EVs could promote cell proliferation and angiogenesis via Akt and Erk pathways, enhance osteogenesis‐related protein expression via the Wnt/β‐catenin signalling pathway, inhibit cell apoptosis via the Akt/Bad/Bcl‐2/Caspase‐3 signalling pathway and finally prevent and treat hormonal ischemic necrosis of the femoral head.^65^ Despite these encouraging preclinical results, the underlying tissue‐regeneration mechanisms still need to be confirmed for future clinical applications of PRP‐EV‐based therapies, as briefly discussed here (Figures 1 and 3).

**TABLE 2:** Some preclinical applications of PRP‐EVs in regenerative medicine

Investigators and reference	Diseases	Animal model	Amount of administered PRP‐EVs	Underlining mechanisms and results
Brill et al. (2005)^102^	Chronic myocardial ischemia	Rat myocardial infarction model	Platelet‐derived microparticles (250 μg/ml protein totally)	Platelet‐derived microparticles improve the revascularization after chronic ischemia
Li et al. (2021)^128^	Myocardial ischemia‐reperfusion	Mouse model of myocardial ischemia‐reperfusion (MI/R)	Platelet‐mimetic EVs (100 μg per mouse), every 7 days for up to 4 weeks	Engineering platelet extracellular vesicles enhance the angiogenesis potency
Ma et al. (2021)^129^	Atherosclerosis	ApoE‐KO mouse model	Platelet‐derived EVs (10 mg/kg)	Platelet‐derived extracellular vesicles loading with MCC950 reduce the formation of atherosclerotic plaques, lower the local inflammation, and inhibit proliferation of macrophages and T cells
Yao et al. (2019)^130^	Atherosclerosis	ApoE−/−high‐fat diet mice	Platelet exosomes (100 nM, every other day)	Platelet‐derived exosomes overexpressing miR−25‐3p attenuate inflammation
Mause et al. (2010)^131^	Vascular injury	Murine model of arterial wire‐induced injury	Angiogenic early outgrowth cells with platelet microparticles (30 μg protein/ml)	Platelet microparticles boost the potential of angiogenic early outgrowth cells to restore endothelial integrity
Lopez et al. (2019)^80^	Haemorrhagic shock	Rat model of uncontrolled bleeding	7.8 × 10^9^ platelet‐derived EVs resuspended in 3 ml of PBS +2 ml of PBS to flush the line	Platelet‐derived extracellular vesicles improve the outcome following severe trauma by maintaining hemodynamic stability and attenuating the development of ischemia, base deficit, and cardiovascular shock
Hayon et al. (2012)^103^	Cerebral ischemia (stroke)	Rats of permanent middle cerebral artery occlusion	Available biodegradable polymer with platelet‐derived microparticles (10 μg/ml or 100 μg/ml)	Platelet‐derived microparticles promote cell proliferation, neurogenesis, and angiogenesis at the infarct boundary zone and significantly improved behavioural deficits
Guo et al. (2017)^66^	Chronic cutaneous wounds	Full‐thickness skin defects in diabetic rat model	Not mentioned	Platelet‐rich plasma‐derived exosomes contribute to angiogenesis through activation of Erk and Akt signalling pathways, and re‐epithelialization via activation of YAP
Xu et al. (2018)^132^	Diabetic skin wounds	Full‐thickness skin defects in diabetic rat model	Chitosan/silk hydrogel containing 100 μg PRP exosomes	Platelet‐rich plasma‐derived exosomes accelerate wound contraction, re‐epithelialization, collagen synthesis and deposition, along with dermal angiogenesis, thus resulting in faster wound healing
Tao et al. (2017)^65^	Osteonecrosis of the femoral head	Rats with steroid‐induced osteonecrosis of the femoral head	100 μg PRP‐derived exosomes	Platelet‐rich plasma‐derived exosomes have the capability to prevent cell apoptosis in osteonecrosis of the femoral head by promoting Bcl‐2 expression via the Akt/Bad/Bcl‐2 signal pathway
Liu et al. (2019)^133^	Osteoarthritis	Osteoarthritis rabbit model	100 μg/ml PRP‐derived exosomes once a week	Platelet‐rich plasma‐derived exosomes repair osteoarthritis by activating the Wnt/β‐catenin signalling pathway
Ma et al. (2020)^122^	Acute lung injury	Acute lung injury mice	12.6 mg/kg platelet‐derived EVs	Platelet‐derived extracellular vesicles loading with TPCA‐1 reduce the cytokine storm syndromes

### 5.1 PRP‐EVs for procoagulant activity and haemostasis

It is well known that platelets play an important physiological role in haemostasis and coagulation.^93^ For trauma patients with severe bleeding, haemostasis is the first as well as a very critical physiological process necessary to prevent and treat the acute injury. However, compared with platelets, PRP‐EVs seem to be a superior alternative for local coagulation and haemostasis following injury. A study by Sinauridze et al. showed that the surface of the PRP‐EVs is ~50‐ to 100‐fold more procoagulant than that of the activated platelets.^68^ Many coagulation‐related materials are loaded in PRP‐EVs, including prothrombotic proteins annexin‐V, factor X and prothrombin, which significantly improve the procoagulant function of PRP‐EVs.^94^, ^95^, ^96^ Another reason for the increased coagulation ability is an increase in the area exposing PS on the surface of the PRP‐EVs, which is a negatively charged molecule on the intracellular surface of the platelet.^94^, ^96^, ^97^

Dyer et al. found that trauma could result in a significant release of platelet‐derived EVs, which cause an increase in thrombin generation and platelet aggregation, as well as a decrease in bleeding time and uncontrolled haemorrhage.^98^ Lopez et al. also demonstrated that PRP‐EVs could provide prohemostatic support following uncontrolled haemorrhage and improve the prognosis via maintaining hemodynamic stability and weakening the development of ischemia, base deficit and cardiovascular shock. Furthermore, they recommended PRP‐EVs as an alternative therapy for providing haemostasis for trauma patients.^80^ Some reviews have also discussed the advantages and related mechanisms of PRP‐EVs as suitable alternatives for coagulation and haemostasis.^23^, ^97^, ^99^ A review conducted by Johnson et al. suggested that, compared with the traditional platelet transfusion approach, PRP‐EVs could retain their functions after the freeze–thaw cycles, thus potentially overcome the current limitations of storing, transporting and using fresh platelets. Therefore, at least under some specific conditions, PRP‐EVs seem to be a superior candidate to restore haemostasis and inhibit vascular permeability.^83^

However, with regard to the local administration of PRP‐EVs as a therapeutic tool, the pro‐coagulant activity of PRP‐EVs seems to be a barrier. Puhm et al. suggested that the effects of PRP‐EVs were different depending on the trigger of PRP‐EVs generation.^99^ For example, PRP‐EVs derived from resting platelets showed milder pro‐coagulant and haemostatic properties than that derived from thrombin‐activated platelets.^99^ Therefore, to meet the purpose of local administration, PRP‐EVs derived from specific trigger is necessary to reduce the pro‐coagulant activity.

### 5.2 PRP‐EVs in angiogenesis

Angiogenesis is an integral process in regenerative medicine. Newly formed vascular networks are helpful for the successful delivery of nutrients and oxygen and for the removal of wastes from lesion sites, maintaining high metabolic activity for tissue repair and regeneration. At present, the positive effects of PRP on angiogenesis are well accepted. The high concentrations of growth factors and cytokines from PRP significantly contribute to angiogenesis and vasculogenesis.^15^

More interestingly, PRP‐EVs have also been demonstrated to play an important role in angiogenesis and endothelial regeneration. In in vitro studies, Kim et al. first demonstrated that PRP‐EVs can promote the proliferation, survival, migration and tube formation in human umbilical vein endothelial cells (HUVECs) via the pertussis toxin‐sensitive G protein, extracellular signal‐regulated kinase and PI3K pathway.^100^ Sun et al. also demonstrated that activated PRP‐EVs could promote the proliferation and migration in HUVECs and the high expression of miR‐126 and angiogenic factors.^101^ In in vivo studies, the functions on angiogenesis and postischemic revascularization were also confirmed in PRP‐EVs.^102^, ^103^ Tao et al. similarly found that PRP‐EVs promote the repair and regeneration of bone tissue after osteonecrosis of the femoral head via inducing local angiogenesis.^65^ Guo et al. also demonstrated that one of the important mechanisms of PRP‐EVs in promoting the re‐epithelization of chronic cutaneous wounds is local angiogenesis.^66^

The great potential of PRP‐EVs in angiogenesis is well accepted, but opinions on the relevant mechanisms remain controversial. It is generally accepted that some proangiogenic growth factors play an indispensable role in angiogenesis, such as VEGF, PDGF, bFGF, IGF‐1, EGF and TGF‐β1.^104^ Torreggiani et al. compared the concentration of important growth factors in PRP and PRP‐EVs and found that a higher amount of bFGF, VEGF, PDGF‐BB and TGF‐β1 was present in PRP‐EVs.^64^ Furthermore, Tao et al. and Guo et al. also demonstrated the higher expression of PDGF‐BB, TGF‐β, bFGF and VEGF in PRP‐EVs through western blotting.^65^, ^66^ These results explain the better performance of PRP‐EVs in angiogenesis compared to PRP. Moreover, another potential mode of proangiogenic response is the mediation of MMPs in vascular endothelial cells. Previous studies have demonstrated that PRP‐EVs can improve the expression levels of MMP‐2 and MMP‐9 in HUVECs, although these enzymes are not included in PRP‐EVs.^105^ Overall, the excellent capability in angiogenesis is an important mechanism of PRP‐EVs in promoting tissue repair and regeneration.

### 5.3 The pro‐inflammatory and anti‐inflammatory properties of PRP‐EVs

As well as their important roles in haemostasis and angiogenesis, PRP‐EVs have prominent pro‐inflammatory and anti‐inflammatory properties. In fact, PRP‐EVs have been traditionally regarded as powerful pro‐inflammatory mediators. For example, some scholars believe that PRP‐EVs may mediate the inflammatory reaction following some cases of platelet transfusion.^106^, ^107^ PRP‐EVs directly cause inflammation after infection through recruiting T cells, B cells and monocytes and enhance the interaction between monocytes and endothelial cells through the binding of P‐selectin and PSGL1.^108^ Moreover, some inflammation‐related molecules, such as lipid mediators, IL‐1β and damage‐associated molecular patterns, are rich in PRP‐EVs, indicating the positive effects of PRP‐EVs on the transfer of inflammatory signals.^84^, ^109^, ^110^, ^111^

On the contrary, some scholars also suggest that PRP‐EVs may contribute to anti‐inflammatory effects. For example, PRP‐EVs may reduce the inflammatory reaction via inhibiting the production of pro‐inflammatory factors, such as TNF‐α from macrophages^88^ or TNF‐α and IL‐8 from plasmacytoid dendritic cells.^112^ Moreover, EVs from stored PRP can polarize macrophages and transfer them to an anti‐inflammatory phenotype.^88^ PRP‐EVs can also improve the production of lipoxin A4 to resolve inflammation through providing 12‐lipoxygenase to mast cells.^113^ A review conducted by Puhm et al. confirmed that the different roles of PRP‐EVs in inflammation might be due to their different subtypes activated by different agonists.^99^ However, it seems to be difficult to enhance a specific inflammatory function and inhibit the other contrary inflammatory function during the applications of PRP‐EVs. Future studies should explore more possible solutions to avoid the unpredictable pro‐inflammatory properties of PRP‐EVs during the clinical applications. The pro‐inflammatory and anti‐inflammatory properties of PRP‐EVs individually influence tissue repair and regeneration following degenerative diseases and secondary injuries after trauma.

### 5.4 PRP‐EVs influence the biological behaviours of cells

Tissue proliferation to repair or replace damaged tissues is an essential phase in the process of tissue repair and regeneration after the haemostasis and inflammation phases. During this phase, the basic structural foundation for the restoration of functions in damaged tissues is established via promoting the biological behaviours of cells, including survival, proliferation, migration and differentiation. Evidence of the beneficial properties of PRP‐EVs for multiple cell types is increasing. As discussed in the above section, PRP‐EVs therapy can significantly enhance the biological behaviours of vascular endothelial cells including the recruitment, proliferation, migration and tube formation capacity, as well as promote local angiogenesis.

In other cell types, PRP‐EVs also show a powerful intervening capacity. Guo et al. found that PRP‐EVs could greatly increase the proliferation and migration of primary dermal fibroblasts through YAP de‐phosphorylation, increase CTGF secretion and accelerate wound healing.^66^ Similarly, Tao et al. demonstrated that PRP‐EVs could effectively inhibit apoptosis and promote the proliferation and osteogenic differentiation of murine osteoblastic MC3T3‐E1 cells and MSCs.^65^ Moreover, PRP‐EVs may help in influencing the biological behaviours of nerve cells. Studies have indicated that the administration of PRP‐EVs leads to the combined augmentation of neurogenesis and angiogenesis and results in improved functional gain after a stroke.^103^

More importantly, maintaining the populations of stem cells and reinforcing their biological functions in lesion sites seem to be more meaningful for tissue repair and regeneration. PRP‐EVs exhibit excellent regulatory properties for multiple types of stem cells. For instance, in terms of MSCs, several studies have confirmed that PRP‐EVs can promote the survival, growth, migration and directional differentiation to a greater extent.^64^, ^65^, ^114^, ^115^ Moreover, in terms of neural stem cells, PRP‐EVs are also suggested to enhance cell proliferation and survival and increase the potential for differentiation to neuroglia and neurons.^116^ The abundant proteins, lipids and nucleic acids in PRP‐EVs make it convenient for them to influence the biological behaviours of various cells. However, to realize their full therapeutic potential in regenerative medicine, identifying the key molecular players and elucidating their mechanisms are necessary.

### 5.5 PRP‐EVs as delivery vehicles

With the development of precision medicine, EVs, as delivery vectors, have attracted a wide range of attention in the field of tissue engineering and regenerative medicine. The phospholipid bilayer structures providing sufficient protection for loaded cargoes and the membrane integrins and receptors inherited from parental platelets providing potential targeting ability make PRP‐EVs ideal candidates for the targeted repair of tissues, especially vascular and inflammatory tissues.^83^ Moreover, the nanoscale size of PRP‐EVs is likely to contribute to their stability in circulation and the possibility of transfer across biological barriers such as the blood–brain barrier. In general, there are two types of cargoes exchanged with other cells via PRP‐EVs: endogenous and exogenous cargoes.

On the one hand, PRP‐EVs load endogenous cargoes from parental platelets to influence the functions of target cells. As discussed in the above section, large amounts of growth factors including bFGF, VEGF, PDGF‐BB and TGF‐β1 are rich in PRP‐EVs to promote tissue repair and regeneration. In addition, many miRNAs from platelets, such as miRNA‐24,^117^ miRNA‐223^118^, ^119^ and miRNA‐126,^119^ are loaded in PRP‐EVs and incorporated into target cells, which can lead to various effects.^120^ Interestingly, mitochondria can also be released from platelets as a kind of cargo in EVs.^99^ These mitochondria in PRP‐EVs are functional and may serve as important moderators in reprogramming the metabolism of the recipient.^121^

On the other hand, exogenous drugs are also loaded into PRP‐EVs by functionalization strategies. Currently, although the cargo loading strategies are varied, two main loading strategies are dominant: the endogenous and exogenous cargo loading strategies. The endogenous cargo loading strategy is based on drug preloading in platelets followed by PRP‐EV generation, and the exogenous cargo loading strategy is based on the post‐loading of isolated PRP‐EVs.^83^ Multiple studies have shown that different exogenous drugs can be loaded into PRP‐EVs via different strategies to enhance the specific theranostic effects. For example, Ma et al. designed engineered PRP‐EVs for the targeted delivery of [5‐(p‐fluoro‐phenyl)‐2‐ureido] thiophene‐3‐carboxamide due to the intrinsic capacity to target pneumonia and thereby confirmed their therapeutic benefits in acute lung injury by inhibiting the infiltration of pulmonary inflammatory cells and attenuating local cytokine storms. Meanwhile, they also believed that PRP‐EVs could serve as ideal delivery carriers, which can selectively target various inflammatory sites, including chronic atherosclerotic plaque, rheumatoid arthritis and wounds associated with the skin.^122^ Other studies have also verified the high loading and delivery efficiency for antiviral and antitumor drugs.^123^, ^124^

Overall, the excellent loading capacity of PRP‐EVs opens up more possibilities in regenerative medicine. Moreover, compared with EVs derived from stem cells, a large amount of PRP‐EVs can be directly produced from platelet concentrates, which seem to be a more convenient and feasible candidate as a drug carrier.

## 6 ADVANTAGES AND LIMITATIONS OF PRP‐EVS IN REGENERATIVE MEDICINE

To date, studies have shown the great potential of PRP‐EVs in the field of tissue repair and regeneration and, to some extent, revealed their related mechanisms. Recent findings suggest that PRP‐EVs may be a superior alternative in regenerative medicine, compared to the well‐studied PRP.^64^, ^65^, ^66^, ^83^ Although it remains to be further demonstrated, PRP‐EVs may have more significant advantages over PRP in regenerative medicine for the following reasons: (1) some studies have confirmed the higher concentration of growth factors in PRP‐EVs as compared to PRP (Figure 1)^64^, ^65^, ^66^; (2) the smaller size of PRP‐EVs is beneficial for their transfer across biological barriers and helps to retain stability in the extracellular environment; (3) they represent a subcellular therapeutic strategy with lower immunogenicity; (4) they provide more sufficient protection for loaded cargoes from the phospholipid bilayer structures; (5) abundant information molecules such as proteins, lipids and RNAs in PRP‐EVs contribute to their participation in intercellular communication.^106^, ^125^

Furthermore, as compared with EVs from other sources, especially stem cells, PRP‐EVs show some advantages in several aspects. First, owing to the direct extraction from platelet concentrates, the isolation of PRP‐EVs lowers the requirements of upstream expansion and avoids the critical procedure and quality control issues associated with cell amplification, which is indispensable for EVs from other cells.^50^ Additionally, the platelet concentrate is a kind of essential medicine authorized by the World Health Organization. Blood donation is legal and encouraged in the majority of countries. However, most blood donation is administrated to meet the clinical need for red blood cells; only 20% is collected for the preparation of platelet concentrates, which implies a rich allogeneic platelet source for the isolation of PRP‐EVs.^83^ In some emergency situations, autologous blood is also a very convenient option. However, although PRP‐EVs are derived from platelet concentrates, current regulatory authorities classify EVs as biological medicinal products, thus, their regulatory rules are significantly different from those of blood products.^83^ In future, as other kinds of blood products and EVs, the manufacturing process and therapeutic applications of PRP‐EVs should undergo rigorous scrutiny using newly developed specific regulatory guidelines. Finally, the anucleated property of platelets also decreases safety concerns about possible tumorigenic risks.

However, as a novel subcellular therapeutic strategy in regenerative medicine that has only been studied in depth in recent years, some limitations still exist in the application of PRP‐EVs. First, as mentioned above, a rigorous complex regulatory guideline for the manufacturing process and therapeutic applications of PRP‐EVs should be established to develop safe and effective PRP‐EV clinical therapy in future.^83^ Second, more technical challenges during the process of their manufacture and clinical application should be considered, including the most suitable and economical production and isolation methods, the optimal collecting mode, the optimum storage method, their shelf‐life, the recommended dose for clinical efficacy, the optimal mode of administration to avoid rapid clearance, their half‐life and the optimal clinical indications. Moreover, research on the potential mechanism of formation and application of PRP‐EVs remains in the early stages. More basic studies should be focused on revealing the physiological functions of PRP‐EVs with different membrane markers and internal compositions (proteins and RNAs) generated by different triggering mechanisms and distinguishing precisely these two subtypes of PRP‐EVs and defining their different physiological functions. Finally, as with PRP, PRP‐EVs are susceptible to individual variability. One way to maintain the consistency in the quality and efficacy of allogeneic PRP‐EVs is to establish a PRP pooling to alleviate the individual donor variations. The establishment of functional assays *in intro* and animal models *in vivo* which are based on the clinical targeted indications will contribute to determine the batch‐to‐batch consistency and alleviate the influences on biochemical properties of PRP‐EVs. This important information gained from preclinical and clinical studies will contribute to the rapid introduction of PRP‐EVs to clinics in the near future.

## 7 CONCLUSIONS AND FUTURE PROSPECTS

Current studies have gradually demonstrated the same, or even better, performance of PRP‐EVs in the field of tissue repair and regeneration, which indicates that PRP‐EVs may be a superior alternative in regenerative medicine. Based on this, this review concluded the possible mechanisms of PRP‐EVs related to regenerative medicine and the advantages and limitations for future clinical translation. This review has focused on five possible mechanisms concerning regenerative medicine, including procoagulant activity and haemostasis, angiogenesis, pro‐inflammatory and anti‐inflammatory properties, the influence on cell biological behaviours and their role as delivery vehicles. These mechanisms recommend PRP‐EVs as a promising candidate in the regeneration of multiple types of tissues and have brought new hope for the treatment of many degenerative and traumatic diseases.

However, studies regarding the application of PRP‐EVs in general can be said to still be in their infancy, especially in regenerative medicine. There remain many barriers between basic research and clinical application that need to be broken in the future. To develop PRP‐EVs for use as recognized clinical therapeutic techniques, more challenges need to be addressed, including a rigorous complex regulatory guideline for the manufacturing process and therapeutic applications of PRP‐EVs, more technical challenges during the process of their manufacture and clinical application, and a better understanding of the potential mechanism of formation and application of PRP‐EVs. In future, official rigorous regulation, improved standardized techniques and essential information from preclinical and clinical evaluations will enable a comprehensive characterization of PRP‐EVs and further advance our understanding of PRP‐EVs to develop novel PRP‐EVs‐based therapeutic approaches. Nevertheless, we believe that, with the continuous advances in the understanding of the molecular mechanisms of PRP‐EVs and with more convincing clinical evidence, PRP‐EVs may replace the application of PRP or even become a superior alternative for regenerative medicine in the near future.

## CONFLICTS OF INTEREST

All authors declare no conflict of interest.

## AUTHOR CONTRIBUTIONS

Conceptualization, J. Wu, Q. Liu and X. Yang; writing original draft preparation, J. Wu; writing‐review and editing, Y. Piao, Q. Liu and X. Yang. All authors have read and agreed to the published version of the manuscript.

## Data Availability

Data sharing is not applicable to this article, as no new data were created or analysed in this paper.
